# Epidemiological investigation of a food-borne outbreak in a kindergarten, Jeju Province, Korea

**DOI:** 10.4178/epih.e2023047

**Published:** 2023-04-17

**Authors:** Kyoung Mi Kim, Eun Suk Cho, Seong Bae Ahn, Eun Ok Kang, Jong-Myon Bae

**Affiliations:** 1 Jeju Center for Infectious Diseases Control and Prevention, Jeju, Korea; 2 Bureau of Women’s Health Welfare, Jeju, Korea; 3 Jeju Special Self-Governing Province Institute of Health and Environment Research, Jeju, Korea; 4 Department of Preventive Medicine, Jeju National University College of Medicine, Jeju, Korea

**Keywords:** Foodborne disease, *Bacillus cereus*, Disease outbreaks, Infection control

## Abstract

**OBJECTIVES:**

On Monday, September 6, 2021, at a kindergarten in Jeju Province, a large number of children vomited and developed food poisoning symptoms, and this necessitated an epidemiological investigation.

**METHODS:**

The team surveyed symptoms and food intake history of kindergarten children, teachers, and workers who ate lunch between September 2 (Thursday) and September 6 (Monday), excluding weekends. In addition to rectal swabs, environmental samples from preserved foods, cooking utensils, drinking water, and refrigerator handles were collected. Pulsed field gel electrophoresis (PFGE) for genetic fingerprint analysis was also performed.

**RESULTS:**

There were 19 cases among 176 subjects, which indicated an attack rate of 10.8%. The epidemic curve showed a unimodal shape, and the average incubation period was 2.6 hours. While no food was statistically significant in food intake history, the analysis of 35 rectal smear samples detected *Bacillus cereus* in 7 children, 4 teachers, and 1 cooking staff. Enterotoxins were also detected in 12 samples. Out of 38 environmental samples, *B. cereus* and enterotoxins were detected in the morning snack cereal, lunch bean sprouts, and afternoon snack steamed potatoes on Monday, September 6th. The result of the PFGE test on 10 isolates of *B. cereus* showed that there was no genetic homology.

**CONCLUSIONS:**

Our results indicated that this outbreak was simultaneously caused by various strains of *B. cereus* from the environment.

## INTRODUCTION

*Bacillus cereus* is a gram-positive, spore-forming bacterium that produces endotoxins, causing food poisoning outbreaks [1-3]. Foodborne outbreaks caused by *B. cereus* account for 1.4-12.0% worldwide [4]. In Korea, *B. cereus* accounted for 2.7% of the 5,366 foodborne poisoning in 2002-2021 year with 144 cases [5]. *B. cereus* is widely distributed in nature and commonly found in a variety of foods, and it can be isolated from patients’ specimens, including vomit and feces; however, it is difficult to identify it as the causative agent of food poisoning. Because its clinical manifestations are similar to food poisoning from *Staphylococcus aureus* and *Clostridium perfringens* and there are limitations in testing for its toxins, the actual incidence of *B. cereus* infection is likely underreported [1,6].

On Monday, September 6, 2021, at around 5:00 p.m., the director of a kindergarten in Jeju City, Jeju Special Self-Governing Province, suspected a food poisoning outbreak and called the local community health center. The main report was that one child vomited around 3:00 p.m. that day, followed by eight additional children with the same symptoms. Given that many children presented with vomiting over a short period, a food-borne and water-borne outbreak was suspected and an epidemiological investigation was initiated the same day. The objectives of this epidemiological investigation were to determine the extent of the suspected foodborne outbreak, identify the source and causative agent, and establish measures to prevent and control the spread of the outbreak.

## MATERIALS AND METHODS

### Case definition

According to the Guidelines for Control of Water-borne and Food-borne Infectious Diseases of the Korea Disease Control and Prevention Agency (KDCA) [7], the presumptive exposure period for investigating the common food history was set from the meal time on September 2 (Thursday), five days before the outbreak, to September 6 (Monday), the day of the onset of symptoms. Therefore, the case was defined as ‘who had a history of eating at least one meal at the kindergarten between the morning snack on September 2 (Thursday) and the afternoon snack on September 6 (Monday), and who had at least one gastrointestinal symptom (nausea, vomiting, abdominal pain, or diarrhea)’. Pathogen carrier was defined as who had a positive rectal smear test for the infectious agent without subsequent symptoms. This included not only children but also staff and cooks. We conducted a retrospective cohort study of those who met the definitions.

### Collection of data and specimens

A total of 176 subjects, including children, teachers, and cooking staff who had eaten kindergarten meals, were subjected to this investigation. The study erroneously excluded 27 children in the 4-year-old class and 30 children in the 5-year-old class because they did not develop symptoms. On September 6 (Monday), we visited the kindergarten to inquire about the presence of symptoms (such as diarrhea, fever, chills, nausea, vomiting, and abdominal pain), number of diarrhea episodes, diarrhea patterns, current symptoms, and food intake history from September 2 (Thursday) to September 6 (Monday) among classes with symptomatic children based on the Standard Epidemiological Survey Questionnaire for Water-borne and Food-borne Infectious Diseases [7]. For data collection, teachers and cooking staff were asked to fill out a self-administered questionnaire, while the teachers reported the symptoms and food intake history of the children by interviewing their parents.

We also visited the kindergarten to inspect the classroom layout, environment, hygiene, food storage conditions, and food preparation, transportation, and serving processes. In addition, we checked whether there were any cuts or purulent lesions on the hands of the cooking staffs.

Rectal smears were taken from symptomatic individuals and staff, and environmental samples were collected from preserved foods, cooking utensils, drinking water, and refrigerator handles. Thereafter, we requested the Research Institute of Public Health and Environment to perform a pathogen test. Based on the results, pulsed field gel electrophoresis (PFGE) was performed using the PulseNet method, a standardized national surveillance laboratory method, to determine genetic links through genetic fingerprinting. Further genetic characterization was performed by the Division of Bacterial Diseases in KDCA. In addition, different tests were performed on the toxin genes of *B. cereus* as follows: *non-hemolysin (nheA), hemolysin (hblC), enterotoxin FM (entFM), cytotoxin K (cytK)* and *B. cereus toxin (bceT)* as diarrheal toxin genes; and *cereulide (CES)*, an emetic toxin gene.

### Statistical analysis

Attack rates and symptom-specific prevalence are expressed as percentages (%), and 95% confidence intervals (CIs) for attack rates were calculated according to a binomial distribution. As for the incubation period, we calculated the average, minimum-maximum, median, and mode values. Differences by sex, age group, and class were calculated using the chi-square method, and relative risks (RRs) and 95% CIs for each food were calculated using Stata version 15 (StataCorp., College Station, TX, USA). Statistical significance was set at 0.05.

### Ethics statement

This study was approved for exemption from research review by the Institutional Review Board of Jeju National University (JJNU-IRB-2022-007).

## RESULTS

### Demographic characteristics of cases

In a retrospective cohort study, 176 individuals were included, out of which 19 met the case definition, with an attack rate of 10.8% (95% CI, 6.63 to 16.34). All were children including 7 boys and 12 girls, with an attack rate of 9.1% and 12.1%, respectively, indicating no sex difference. The highest attack rate by age group was in the 3-year-olds at 15.9%, followed by 4-year-olds at 11.1% and 5-year-olds at 10.3%. However, there were no significant differences by age group. There were five carriers with positive rectal smear without symptoms, and these included 4 teachers and 1 cooking staff (Table 1). The main clinical symptom was vomiting at least once, which occurred in all except 1 child, followed by nausea in 3 (15.8%), diarrhea in 2 (10.5%), and abdominal pain in 2 (10.5%) (Table 2). Nothing abnormal was observed when the hands of the cooking staffs were examined for cuts and purulent lesions.

### Temporal characteristics of cases

**Figure 1.**
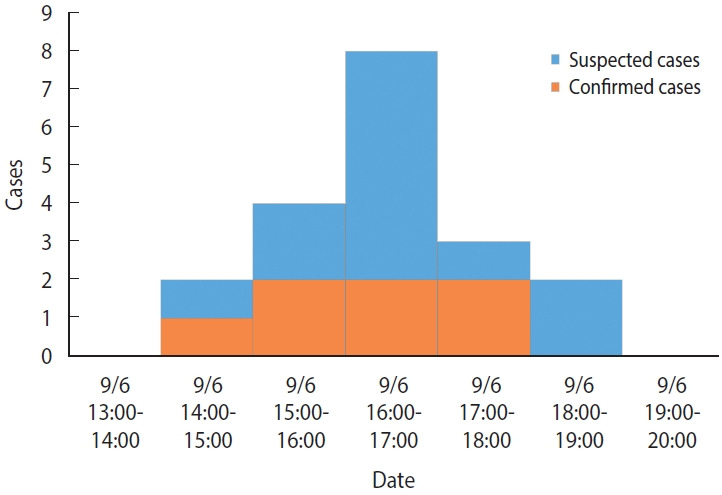
Epidemic curve of the cases.

There were no previous cases before September 6, and the time of symptom onset was 14:00-15:00 for 2 cases (10.5%), 15:00-16:00 for 4 cases (21.1%), 16:00-17:00 for 8 cases (42.1%), 17:00-18:00 for 3 cases (15.8%), and 18:00-19:00 for 2 cases (10.5%) on September 6 (Monday), which indicated a unimodal distribution (Figure 1). The mean incubation period, using the afternoon snack on September 6 (Monday) as a common source of exposure, was 2.6 hours, with minimum, maximum, median, and mode values of 0.8 hours, 4.5 hours, 2.5 hours, and 3.0 hours, respectively.

### Spatial characteristics of cases

The kindergarten building consisted of one semi-basement and one ground floor. The ground floor had a common room, a textbook room, two resource rooms, a director’s office, an administrative office, a teacher’s office, an outdoor restroom, an indoor restroom, and four classrooms, including one for 5-year-old, one for 4-year-old, and two for 3-year-old. The two 3-year-old classes with a total of 22 students each had 2 cases (Class 5) and 5 cases (Class 6) and the one 4-year-old class with a total of 27 students had four cases (Class 4). The remaining 5-year-old were asymptomatic and case-free. The semi-basement floor contained a multipurpose room, a textbook room, a dining room, a kitchen, an outdoor restroom, two indoor restrooms, and four classrooms, including two for 4-year-old and two for 5-year-old. Two cases (Class 3) occurred in one 4-year-old class with a total of 27 members. Two 5-year-old classes with a total of 29 members each had one case (Class 1) and five cases (Class 2), respectively, of which the first indicator child (age 5, girl) case occurred around 14:50 after eating the afternoon snack (Class 2). The remaining 4-year-old class was case-free, and none of the children were symptomatic. Symptoms were observed in six out of a total of eight classes: three on the ground floor and three in the semi-basement.

The kitchen’s ventilation is located directly above the stove and connected to a long duct that leads to the outside. After cooking, the food is cooled on the stove or a shelf in the center of the kitchen before being served. The steamed potatoes that were served as a snack on September 6 (Monday) were steamed in a drum, with the lid removed, followed by cooling on the spot and serving to children.

### Analysis of food intake history

Based on the menu 5 days before the first symptom onset, relative risks were analyzed for the intakes of morning snacks, lunches, and afternoon snacks for the three days — September 2 (Thursday), September 3 (Friday), and September 6 (Monday), excluding weekends, but no significant results were obtained. There were three cases of food with *B. cereus* detected in laboratory tests, which were suspected risk factors. However, the intake history of the food provided had been investigated a while ago. In addition, the subjects were kindergarten children aged 3 years, 4 years, and 5 years old, indicating that the investigation relied on the memory of the teachers who assisted them with meals. Thus, there were limitations in obtaining an accurate food intake history (Table 3).

### Laboratory results of human and environmental specimens

A total of 35 human specimens (18 symptomatic children, 13 teachers, and 4 cooking staffs) were obtained by rectal smear. *B. cereus* was detected in 7 out of the 18 symptomatic children (7 positives for endotoxins), 4 out of 13 teachers, and 1 out of 4 cooking staffs (5 positives for endotoxins). Enteropathogenic *Escherichia coli* was also detected in the specimens from the cooking staff infected with *B. cereus*.

Laboratory tests were performed on 38 environmental specimens (28 preserved foods, 8 cookware, 1 refrigerator handle, and 1 drinking water). A total of 28 specimens of 3-day’ preserved foods were tested for causative agents of food poisoning, which revealed *B. cereus* in the cereal for morning snack, seasoned bean sprouts for lunch, and steamed potato for afternoon snack on Monday, September 6 (3 endotoxin positives). None of the remaining 10 environmental specimens contained any pathogens (Table 4). The cooking water was from the municipal water supply and contained 0.2 ppm of chlorine (normal range, 0.2 to 0.5).

### Pulsed field gel electrophoresis analysis of toxin genes

**Figure 2.**
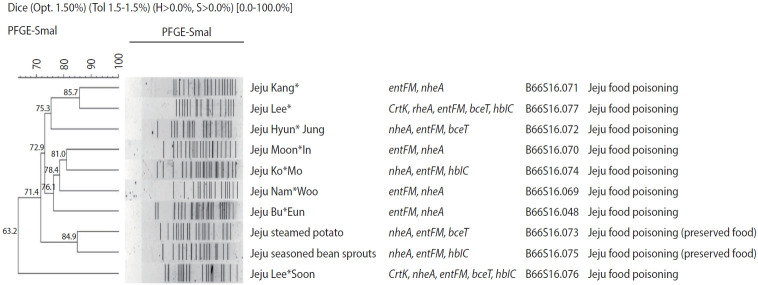
Pulsed field gel electrophoresis (PFGE) banding patterns of *Bacillus cereus. nheA, non-hemolycin; hblC, hemolysin; entFM, enterotoxin FM; cytK, cytotoxin K; bceT, Bacillus cereus toxin; CES, cereulide*.

All the 15 specimens with *B. cereus* were positive for *nheA* and *entFM* in the toxin gene test (Table 5). PFGE testing of *B. cereus* isolates from 5 children, 3 teachers, and 2 samples of preserved foods from environmental samples showed similarities ranging from 85.7% to 63.2% (Figure 2). According to the Pulse Net Korea database, B66S16.048 was the type identified in the isolates of Jeju Seogwipo outbreak in 2018 and B66S16.069-77 was the first type identified in Korea.

## DISCUSSION

The following summarizes the findings from this epidemiologic investigation. First, the infected children exhibited nausea, vomiting, abdominal pain, and diarrhea without fever, which are typical of emetic illnesses. In particular, 94.7% of the primary clinical symptoms was vomiting. Second, the epidemiological curve showed a unimodal shape with concentrated outbreaks over a 5-hour period. The mean incubation period was 2.6 hours, with a minimum and maximum incubation period of 0.8 hours and 4.5 hours, respectively, which was similar to previous reports on the incubation period (1-5 hours) of *B. cereus* infection [1,4]. Third, laboratory testing of human and environmental specimens resulted in the identification of toxin-positive *B. cereus* isolates in seven of the cases and the detection of bacteria toxins in some preserved foods. Based on these main findings, we concluded that this outbreak was food poisoning caused by emetic *B. cereus* infection.

The primary routes of transmission of *B. cereus* are through the ingestion of food that has been cooked and then held at a mild temperature, ingestion of bacteria growing on various parts of vegetables, ingestion of a rice-based food that has been held at a mild temperature before reheating, and consumption of food prepared by symptomatic individuals [7]. While *B. cereus* was identified in three of the preserved foods obtained, the food intake history survey yielded no significant results. This could be attributed to the limitation of the field investigation, where the food intake history of kindergarteners could only be obtained from their teachers.

In this epidemiologic investigation, *B. cereus* was identified in three preserved foods, including cereal, seasoned bean sprouts, and steamed potato. This is consistent with the report that starchy foods are the main traditional food vectors of *B. cereus* [8], and that, particularly, starchy pasta and infant weaning food are more susceptible to emetic *B. cereus* contamination than other foods [9]. During the on-site inspection, the kitchen facilities were inspected, and we found that the food was cooked and cooled on the stove with the lid removed, and the ventilation was located directly above the stove. In particular, it was difficult to naturally ventilate the kitchen due to the semi-basement floor, and there was no proper discharge system for steam. This is consistent with the fact that improper cooling of food during cooking or preserving cooked food at room temperature contributes to the generation of cereulide toxins [10].

Moreover, the genetic test indicated no matches for the toxin types, and PFGE analysis showed no genetic homology, suggesting that the infections were not from the same source of contamination. This indicated that there was no human-to-human transmission in this foodborne outbreak and that the infections were simultaneously caused by various *B. cereus* strains from the environment.

The limitations of this epidemiological study were as follows: First, during the on-site inspection, children in the 4-year-old and 5-year-old cohorts who were eligible but did not develop symptoms were excluded from the study due to miscommunication, and their food intake history was not surveyed. Therefore, the food intake analysis table excluded the data of 57 children who were not surveyed, indicating that the attack rate in this study may be higher than the actual infection rate and the actual distribution of demographic variables may be different. Second, as the epidemiological investigation was conducted among kindergarten children, subjective clinical symptoms and food intake history were identified by relying on the teachers’ memories and checking through parental interviews, which was highly prone to measurement error. This may explain why the food intake history did not yield statistically significant results.

In this outbreak, it was difficult to identify the specific source of infection. However, it is suspected that the kindergarten food was contaminated during the cooking process; hence, it is necessary to manage the cooking process and food storage more carefully. Due to the characteristics of the kindergarten building, the kitchen is located in the basement; therefore, care should be taken to prevent bacterial contamination when cooking food. Cooked food should not be stored at room temperature; moreover, ventilation and room temperature should be carefully controlled. In the future, the standard for kitchens in institutional food services should include a requirement for a better ventilation system above ground rather than below ground. More attention should be paid to the maintenance and disinfection of ventilation duct systems.

## Figures and Tables

**Table 1.** Attack rate by sex, age, group (n=176)

Variables		Total, n (%)	Cases (n=19)	Attack rate (%)	95% CI		p-value
					UL	LL	
Sex							0.520
	Male	77 (43.7)	7	9.1	3.73	17.83	
	Female	99 (56.2)	12	12.1	6.42	20.21	
Age (yr)							0.304
	3	44 (25.0)	7	15.9	6.64	30.06	
	4	54 (30.7)	6	11.1	4.19	22.63	
	5	58 (32.9)	6	10.3	3.89	21.17	
	Adult^1^	20 (11.4)	0	0.0	0.00	16.84	
Group (yr-old)							0.226
	Class 1 (5)	29 (16.5)	1	3.4	0.09	17.76	
	Class 2 (5)	29 (16.5)	5	17.2	5.84	35.77	
	Class 3 (4)	27 (15.3)	2	7.4	0.91	24.29	
	Class 4 (4)	27 (15.3)	4	14.8	4.19	33.73	
	Class 5 (3)	22 (12.5)	2	9.1	1.12	29.16	
	Class 6 (3)	22 (12.5)	5	22.7	7.82	45.37	
	Cooking workers^1^	4 (2.3)	0	0.0	0.00	60.23	
	Teachers^1^	16 (9.1)	0	0.0	0.00	20.59	
Total		176 (100)	19	10.8	-	-	

CI, confidence interval; UL, upper limit; LL, lower limit.

1 Pathogen carriers: 5 adults (1 cooking staff and 4 teachers).

**Table 2.** Symptoms in cases (n=19)

Symptoms	n (%)
Vomiting	18 (94.7)
Diarrhea	2 (10.5)
Abdominal pain	2 (10.5)
Nausea	3 (15.8)

**Table 3.** Relative risk for 3 days’ conservative meals (n=176)

Date menu			Intake		Non-intake		Relative risk (95% CI)
			Total (n)	Cases	Total (n)	Cases	
Water: common drinking water (Barley tea)			142	14	25	5	0.48 (0.20, 1.17)
9/2 (Thu)							
	Morning snack						
		Brown rice long gangjeong	150	19	25	0	6.72 (0.42, 107.83)
	Lunch						
		Soosoo rice	150	19	25	0	4.14 (0.26, 65.62)
		Shrimp soup	159	19	16	0	1.40 (0.28, 6.85)
		Mapa tofu	158	18	17	1	4.42 (0.28, 70.07)
		Seasoned vegetables	158	19	17	0	1.57 (0.32, 7.76)
		Radish kimchi	156	18	19	1	5.54 (0.35, 88.46)
	Afternoon snack						
		Stir-fried Udon	154	19	21	0	4.97 (0.31, 9.15)
9/3 (Fri)							
	Morning snack						
		Banana	156	19	19	0	5.54 (0.35, 88.46)
	Lunch						
		Black rice	154	19	21	0	4.14 (0.26, 65.62)
		Soy bean paste soup	159	19	16	0	4.14 (0.26, 65.62)
		Pork bulgogi	159	19	16	0	4.42 (0.28, 70.07)
		Seasoned cucumber onion	158	19	17	0	1.57 (0.32, 7.76)
		Kimchi	156	18	19	1	4.69 (0.29, 74.58)
	Afternoon snack						
		Rice cake slices/Plum tea	157	19	18	0	4.97 (0.31, 79.15)
9/6 (Mon)							
	Morning snack						
		Milk/Cereal	156	19	19	0	3.09 (0.20, 48.35)
	Lunch						
		Multigrain rice	163	19	12	0	1.61 (0.11, 23.98)
		Beef radish soup	169	19	6	0	0.58 (0.13, 2.63)
		Tuna egg roll	168	18	7	1	0.66 (0.14, 3.03)
		Seasoned bean sprouts/Geobong	167	18	8	1	1.85 (0.12, 27.92)
		Kimchi	168	19	7	0	2.09 (0.14, 31.90)
	Afternoon snack						
		Steamed potato/Citrus juice	167	19	8	0	2.58 (0.17, 40.03)

CI, confidence interval.

**Table 4.** Laboratory test results

Specimen			Test item	Results of examination	B. cereus enterotoxin
Human specimen (total 35 cases)			17 Kinds of bacteria, 5 viruses^1^		
	Symptoms kindergartener			*B. cereus* 7 cases	7 Positive
		Rectal swab 18 cases			
	Staff (teachers 13, cooking workers 4)			*B. cereus* 5 cases (teachers 4, cooking worker 1)	5 Positive
		Rectal swab 17 cases		EPEC 1 case (cooking worker 1)	
Preserved meal			Food poisoning caused bacteria (17)^2^		
	9/6 (Mon) Morning snack			*B. cereus*	Positive
		Cereal			
	9/6 (Mon) Lunch			*B. cereus*	Positive
		Seasoned bean sprouts			
	9/6 (Mon) Afternoon snack			*B. cereus*	Positive
		Steamed potato			
Environmental sample (total 10 cases)			Food poisoning caused bacteria (17)^2^		
	Cookware				
		Vegetable cutting board 2		Negative	Negative
		Meat cutting board 2		Negative	Negative
		Fish cutting board 1		Negative	Negative
		Vegetable knife 2		Negative	Negative
		Meat knife 1		Negative	Negative
	Refrigerator handle 1			Negative	Negative
	Drinking water (Barley tea) 1		*Escherichia coli, Salmonella, Yersinia enterocolica*	Negative	Not examined

*B., Bacillus.*

1 17 kinds of bacteria, 5 viruses: EHEC, ETEC, EIEC, EPEC, EAEC, *Vibrio cholerae, Salmonella typhi, Salmonella paratyphi,* Shigella spp., *Vibrio parahemolyticus, Campylobacter jejuni, Listeria monocytogenes, Yersinia enterocolica, Clostridium perfringens,* Rotavirus, Norovirus (GI, GII), Enteric adenovirus, Astrovirus, Sapovirus.

2 Food poisoning caused bacteria (17): Salmonella, *Staphylococcus aureus, Vibrio parahemolyticus, Vibrio cholerae, Vibrio vulnificus, Listeria monocytogenes,* EHEC, ETEC, EIEC, EPEC, EAEC, *B. cereus,* Shigella, *Yersinia enterocolica, Campylobacter jejuni, Campylobacter coli, Clostridium perfringens*.

**Table 5.** Toxin genes and enterotoxin production of isolated *Bacillus cereus*

Isolates	Toxin gene					
	*nheA*	*hblC*	*entFM*	*cytK*	*bceT*	*CES*
Child-1	+	−	+	−	−	−
Child-2	+	−	+	−	−	−
Child-3	+	−	+	−	−	−
Child-4	+	−	+	−	−	−
Child-5	+	−	+	+	−	−
Child-6	+	+	+	−	−	−
Child-7	+	+	+	+	+	−
Teacher-1	+	−	+	−	−	−
Teacher-2	+	+	+	+	+	−
Teacher-3	+	−	+	+	+	−
Teacher-4	+	−	+	−	+	−
Cooking worker-1	+	+	+	−	+	+
Food-1	+	−	+	−	−	+
Food-2	+	+	+	−	−	−
Food-3	+	−	+	−	+	−

*nheA, non-hemolycin; hblC, hemolysin; entFM, enterotoxin FM; cytK, cytotoxin K; bceT, Bacillus cereus toxin; CES, cereulide*.

## SUPPLEMENTARY MATERIALS

Korean version is available at https://www.e-epih.org/.
