# Supplementary material for: Epidemiological investigation of a food-borne outbreak in a kindergarten, Jeju Province, Korea
Source: Epidemiol Health. 2023 Apr 17;45:e2023047. doi: 10.4178/epih.e2023047 (PMC10593584; doi:10.4178/epih.e2023047)
Supplement: Supplementary file 1 [file epih-45-e2023047-Korean-Supplementary.docx]

**<Epidemiologic Investigation>**

**Epidemiological Investigation of a Food-borne Outbreak in a Kindergarten**

**(1개 유치원에서 발생한 식중독 역학조사)**

김경미^1)^, 조은숙^1)^, 안성배^2)^, 강은옥^3)^, 배종면^1,4)^

1. 제주감염병관리지원단, ^2)^ 여성건강복지국, ^3)^제주보건환경연구원, ^4)^제주대학교 의과대학 예방의학교실, 제주특별자치도, 대한민국

교신저자: 배종면

제주도 제주시 제주대학로 102, 63243

이메일: jmbae@jejunu.ac.kr

**Introduction**

바실러스 세레우스 (*Bacillus cereus)*는 그람 양성, 포자형성 세균 (gram-positive, spore-forming bacterium)으로, 장독소 (enterotoxins)를 생성하여 식품매개 식중독 (food poisoning) 집단발생을 야기한다 [1-3]. B. *cereus*에 의한 식품매개 식중독 발생은 전 세계적으로 1.4%~12%를 차지하며 [4], 국내는 2002-2021년도 식중독 발생 5366건 중 바실러스 세레우스는 144건으로 2.7%를 차지하였다 [5]. 그러나 B. *cereus*는 자연계에 널리 분포되어 있고 각종 식품에서 흔히 검출될 뿐만 아니라 환자 검체 중 구토물이나 변에서도 분리되기 때문에 식중독의 원인균으로 동정하기란 쉽지 않다. 더군다나 황색포도상구균 (*Stapholococcus aureus*)과 클로스트리디움 퍼프린젠스 (*Clostridium perfringens*)의 독소형 식중독과 임상 증상이 유사하며, 바실러스 세레우스의 독소 검사법의 한계로 인하여 실제 발생보다 낮게 신고 될 가능성이 높다 [1,6].

2021년 9월 6일 월요일 오후 5시경 제주특별자치도 제주시 소재 모 유치원 원장이 식중독 발생을 의심하여 관내 보건소로 유선 신고하였다. 주된 신고내용은 원아 1명이 당일 오후 3시경 구토 증상을 보인 후, 연이어 원아 8명이 추가로 같은 증상을 보였다는 것이다. 이에 짧은 시간 동안 다수의 원아에서 구토 증상을 보였다는 점에서 식품식수매개 식중독 집단 발생을 의심하고 당일 날 역학조사를 시작하였다. 본 역학조사의 목적은 의심되는 식중독 집단 발생의 규모, 감염원 및 원인체 규명, 그리고 확산방지 및 관리대책 수립하는 것이다.

**Methods**

*사례 정의*

질병관리청의 수인성 및 식품매개 감염병 관리지침 [7]에 따라, 공통 섭취 음식력을 조사하기 위한 추정폭로 기간을 발생 5일 전인 9월 2일(목) 급식 이후부터 증상자 발생 당일인 9월 6일(월)까지로 정하였다. 따라서 역학조사의 사례는 ‘해당 유치원에서 9월 2일(목) 오전 간식부터 9월 6일(월) 오후 간식까지 원내 식사 취식력 1회 이상 있으면서, 1회 이상의 위장관 증상(구역, 구토, 복통, 설사)을 보인 자’이며, 이후 증상 없이 직장도말 검사에서 감염병병원체 양성인 경우는 병원체 보유자 (pathogen carrier)로 정의하였다. 이에 원아뿐만 아니라 직원 및 조리종사자도 포함되었다. 이상의 정의에 따라 후향적 코호트 연구를 수행하였다.

*자료 수집 및 검체 채취*

전체 대상자 233명 중 유증상자가 발생하지 않아 착오로 조사대상에서 제외된 4세분반 27명과 5세분반 30명을 제외한 9월 6일(월) 해당 유치원을 방문하여 9월 2일(목)부터 9월 6일(월)까지 유증상 원아들이 있는 반 중 급식을 섭취한 원아, 교직원, 조리종사자 중 설문에 응답한 176명을 대상으로 표준 수인성‧식품매개 감염병 역학조사서 [7]에 근거하여 증상 유무(설사, 발열, 오한, 매스꺼움, 구토, 복통, 기타), 설사 횟수, 설사 양상, 현재 증상 여부 및 식품 섭취력을 알아보고자 하였다. 자료수집 방법은 직원 및 조리 종사자들에게는 자기 기입식으로 작성하게 하였으며, 원아들은 담당 교사가 학부모와 면담을 통해 증상 및 식품섭취력을 작성하도록 하였다.

또한 현장을 방문하여 해당 유치원의 교실 배치, 환경, 위생 상태 및 식자재 보관상태, 음식 조리과정, 이송과정, 배식 과정 등 전반적인 사항을 점검하였다. 추가로 조리 종사자의 손의 상처 여부, 화농성 병소 여부를 확인하였다.

이후 유증상자 및 직원들에게 직장도말검사를 시행하였고, 보존식, 조리도구, 음용수, 냉장고 손잡이 등에 대한 환경검체를 채취하여 보건환경연구원에 병원체 검사를 의뢰하였다. 그 결과에 따라 유전자 지문분석을 통해 유전학적 연관관계의 확인을 위하여 표준화된 국가감시 실험실 방법인 PulseNet 방법으로 PFGE (Pulsed field gel electrophoresis)를 실시하였으며 유전자 특성 분석은 질병관리청 세균분석과에 의뢰하여 추가로 수행되었다. 또한 바실러스 세레우스의 독소 유전자 중 non-hemolycin (nheA), hemolysin (hblC), enterotoxin FM (entFM), cytotoxin K (cytK), bacillus cereus toxin (bceT)의 설사형 독소 유전자와 cereulide (CES) 구토형 독소 유전자에 대하여 검사가 시행되었다.

*통계분석*

사례 발병률 (attack rate)과 증상별 발현율은 백분율 (%)로 산출하고 이의 95% 신뢰구간 (CI)은 이항분포 (binomial distribution)에 따라 산출하였다. 잠복기는 평균, 최소-최대 그리고 중앙값과 최빈값을 산출하였다. 성별, 연령대별, 반별 차이는 카이자승법을 적용하였으며, 음식별 상대위험도 (relative risk, RR)와 95% 신뢰구간 (CI)은 Stata 15 (StataCorp, Texas, USA)를 활용하여 산출하였다. 통계적 유의수준은 0.05로 정하였다. 본 연구는 제주대학교 생명윤리위원회로부터 연구심의 면제 승인을 받았다 (JJNU - IRB - 2022 - 007).

**Results**

*사례의 인적 특성*

후향적 코호트 조사 대상자 176명에 대한 설문조사를 수행하였고, 이중 사례 정의에 맞는 유증상자는 총 19명으로 발병률은 10.8% (95% CI: 6.63-16.34%)이었다. 모두 원아들이었으며, 남아 7명, 여아 12명으로 성별 발병률은 각각 9.1%, 12.1%로 성별로 차이가 없었다. 연령대별 발병률은 만 3세에서 15.9%로 가장 높았고, 만 4세 11.1%, 만 5세 10.3% 순이었다. 그러나 연령대별로 유의한 차이는 없었다. 증상이 없고 직장도말 검사상 양성인 병원체 보유자는 교직원 4명, 조리 종사자 1명으로 총 5명이었다 (Table 1). 주된 임상 증상으로는 1회 이상의 구토로 1명을 제외한 18명에서 발생하였으며, 오심 3명 (15.8%), 설사 2명 (10.5%), 복통 2명 (10.5%) 순이었다 (Table 2). 조리 종사자의 손의 상처 여부, 화농성 병소 여부 조사를 하였을 때 특이사항은 없었다.

*사례의 시간적 특성*

9월 6일 이전에는 선행 환자가 없었고, 증상 발생 시각은 9월 6일(월) 14:00~15:00 2명 (10.5%), 15:00~16:00 4명 (21.1%), 16:00~17:00 8명 (42.1%), 17:00~18:00 3명 (15.8%), 18:00~19:00 2명 (10.5%)이 발생하여 단봉성의 분포를 보였다 (Figure 1). 이에 9월 6일(월) 오후 간식을 공통폭로원으로 간주하여 잠복기를 산출하였을 때, 평균 2.6시간, 최소 0.8시간, 최대 4.5시간이었으며 중앙값 2.5시간, 최빈값은 3시간이었다.

*사례의 공간적 특성*

유치원 건물은 반 지하 1층과 지상 1층으로 이뤄져 있었고 지상 1층에는 휴게실, 교재실, 자료실 2곳과 원장실, 행정실, 교무실, 외부 화장실 1개, 내부 화장실 1개가 있으며 보통 교실 4개는 5세 분반 1개, 4세 분반 1개, 3세 분반 2개였다. 이중 각각 총 22명 인원인 3세 분반 2곳에서 반별 2명 (Class 5), 5명 사례 (Class 6)가 발생했고 총 27명 인원 4세 분반 1곳에서 4명 (Class 4)의 사례가 발생하였다. 나머지 5세 분반에서는 유증상 및 사례 발생이 없었다. 반 지하 1층은 다용도실, 교재실, 식당과 조리실, 외부 화장실 1개와 내부 화장실 2개가 있었으며 보통 교실 4개는 4세 분반 2개, 5세 분반 2개였다. 총 27명 인원 4세 분반 1곳에서 2명 (Class 3)의 사례가 발생하였고 이중 각각 총 29명 인원인 5세 분반 2곳에서 반별 1명 (Class 1), 5명 (Class 2) 사례가 발생했고 그 중 5명 사례가 발생한 5세반 (Class 2)에서 오후 간식 섭취 후 14:50분경 최초 지표 환아 (만 5세, 여)가 발생하였다. 나머지 4세 분반에서는 유증상 및 사례 발생이 없었다. 전 층 8개 반중 6개 반에서 유증상이 발생하였고 1층 3곳, 반 지하 1층 3곳이었다.

주방의 환기통은 가스레인지 바로 위에 위치하며 긴 관으로 연결되어 외부로 배출되는 형태였다. 음식을 조리하고 나서 가스레인지 위, 혹은 중앙의 선반 위에서 식힌 후 배식을 하게 되며, 9월 6일(월) 오후 간식으로 제공되었던 찐 감자도 드럼통에서 찐 후 뚜껑을 열고 그 자리에서 식힌 후 각 반으로 배식한 것을 확인하였다.

*식품섭취력 분석 결과*

최초 유증상 발생 5일 전 식단을 기준으로 주말을 제외한 9월 2일(목), 9월 3일(금), 9월 6일(월) 3일 동안 오전 간식, 점심, 오후 간식 섭취에 대해 발병률 및 상대위험도를 분석하였으나 유의한 결과를 얻을 수 없었다. 실험실 검사에서 바실러스 세레우스가 검출된 식단이 3건으로 위험요인 의심 식단이었지만 제공된 음식에 대한 섭취력 조사가 어느 정도 시간이 지난 상태이고 조사 대상자가 만 3, 4, 5세의 유치원 원아들이라 옆에서 식사 보조를 했던 선생님의 기억에 의존하여 조사를 진행해 정확한 식품섭취력 파악에 한계가 있었다 (Table 3).

*인체 및 환경 검체의 실험실 검사 결과*

직장도말검사로 총 35건 (유증상 원아 18명, 교직원 13명, 조리 종사자 4명)의 인체검체가 확보되었다. 유증상 원아 18명 중 7명에서 바실러스 세레우스가 검출되었다 (장독소 양성 7명). 교직원 13명 중 4명, 조리 종사자 4명 중 1명에서 바실러스 세레우스가 검출되었다 (장독소 양성 5명). 바실러스 세레우스가 검출된 조리 종사자는 장병원성대장균 (*Enteropathogenic Escherichia coli,* EPEC)도 같이 검출되었다.

환경검체 38건 (보존식 28건, 조리도구 8건, 냉장고 손잡이 1건, 음용수 1건)에 대한 실험실 검사가 이루어졌다. 3일 치 보존식 28건의 식중독 원인균 검사 결과 9월 6일(월) 오전 간식 시리얼, 점심 숙주나물, 오후 간식 찐 감자에서 바실러스 세레우스가 검출되었다 (장독소 양성 3건). 나머지 환경검체 10건에서는 모두 균이 검출되지 않았다 (Table 4). 조리용수는 상수도를 이용하고 있었으며 잔류염소 측정 결과 0.2ppm (정상 범위 0.2-0.5ppm)이었다.

*PFGE 및 독소 유전자 분석 결과*

바실러스 세레우스가 동정된 15건의 검체에 대하여 독소 유전자 검사 결과는 모두에서 nheA와 entFM이 양성이었다 (Table 5). 인체검체 중 원아 5건, 교직원 3건, 환경검체 중 보존식 2건에 대한 바실러스 세레우스 분리주 대상 PFGE 검사 결과, 유사도는 85.72~63.23% 범위로 나타났다 (Figure 2). Pulse Net Korea Database 확인 결과, B66S16.048은 2018년 제주 서귀포 집단발생 분리 병원체에서 확인된 유형이며, B66S16.069~77은 국내 처음으로 확인되는 유형이었다.

**Discussion**

이번 역학조사를 통해 알아낸 결과들을 요약하면 다음과 같다. 첫째, 사례들은 발열이 없고 구토형의 주증상인 오심, 구토, 복통, 설사 증상을 보였다. 특히 주된 임상증상의 94.7%가 구토였다. 둘째, 5시간 동안 집중된 단봉 발생 양상의 유행역학 곡선 형태를 보였으며, 평균 잠복기는 2.6시간, 최소 잠복기 0.8시간, 최대잠복기 4.5시간으로 일반적으로 알려진 바실러스 세레우스 감염증의 구토형 잠복기 (1-5시간)와 유사하였다 [1,4]. 셋째, 인체 및 환경 검체의 실험실 검사 결과 전체 사례 중 7명이 독성 양성인 바실러스 세레우스 균 분리 동정 및 일부 보존식에서 독성 양성 균이 검출되었다. 이상의 주요 결과들에 근거할 때, 이번 집단발생은 구토형 바실러스 세레우스 감염증에 의한 식중독으로 결론지을 수 있겠다.

바실러스 세레우스의 주된 전파경로는 음식 조리 후에 은은한 온도가 유지되면서 끓여지는 온도에서 살아남은 균이 증식하거나 채소의 다양한 부분에서 증식된 것을 섭취한 경우 혹은 집단발생 사례로 쌀을 이용한 요리를 재가열하기 전에 은은한 온도가 유지될 때 구토가 많이 발생하고 증상자가 조리한 요리를 섭취한 경우 등으로 전파된다 [7]. 확보한 보존식중 3가지 음식에서 B. *cereus*가 동정되었지만, 식품섭취력 조사에서는 유의한 결과를 보여주지 않았다. 이는 원아들의 식품섭취력을 담당 교사로부터 얻어낼 수 밖에 없는 현장의 한계 때문으로 해석이 가능하다.

본 역학조사에서 보존식 중에서 B. *cereus*가 동정된 3가지 식품은 시리얼, 숙주나물, 찐 감자였다. 이는 기존의 주요 식품 매개체인 전분 식품이며 [8] 특히 구토형 바실러스 세레우스의 주요 오염 식품으로 전분 성분의 파스타, 유아 이유식이라는 사실과 상응하는 결과이다 [9]. 또한 현장 조사시 주방 시설을 점검하였을 때 음식을 조리한 뒤 뚜껑을 열고 가스레인지 위에서 식히는데, 가스레인지 바로 위에 환기통이 위치하였음을 확인하였다. 특히 반지하 1층에 의하여 자연환기가 어렵고 수증기 배출이 잘 안 되는 환경이었다. 이는 조리 중 음식을 잘못 식히거나 조리된 요리를 실온에서 보존하는 것이 독소 (cereulide)생성의 원인이 된다는 점을 지지하는 것이었다 [10].

한편 독소형 유전자 검사에서 독소형이 일치하지 않았으며, PFGE 분석결과 서로 유전적 상동성은 없는 것으로 나왔다. 이런 소견은 동일한 오염원에 의해 감염된 것이 아니라는 의미이다. 따라서 이번 식중독 집단 발생에서 사람 간 전파가 없었으며, 환경으로부터의 다양한 B. *cereus*가 동시에 오염되어 발생했다고 해석할 수 있다.

이번 역학 조사의 한계점은 다음과 같다. 첫째, 현장 조사를 수행하면서 코호트 조사대상에 해당하지만 유증상자가 발생하지 않았던 4세 및 5세 두 분반에 소속된 원아들에 대하여 의사소통의 착오로 조사대상에서 배제되어 식품섭취력 조사가 이루어지지 않았다. 따라서 식품섭취 분석표는 미조사자 57명의 자료가 제외된 결과이다. 이에 따라 결과에서 산출한 발병률은 실제보다 높게 산출되었으며, 인적 변수의 분포가 실제로는 다를 수 있겠다. 둘째, 유치원 원아를 대상으로 한 역학조사를 수행함에 따라 주관적 임상 증상 여부와 식품섭취력을 담당교사의 기억에 의존해 개별 파악하고 학부모 면담을 통해 확인함으로써 측정오류가 개입될 소지가 높았다. 따라서 식품섭취력에서 통계적으로 유의한 결과가 도출되지 않은 이유가 된다.

이번 집단발생의 경우 특정 감염원을 파악하기 어려웠으나 유치원 식자재의 조리과정 오염을 의심하는 상황이므로 조리과정과 음식물 보관방법에 대한 보다 세심한 관리가 필요하다. 해당 유치원 건물 특성상 지하에 위치한 조리실은 음식을 조리할 때 균이 오염되지 않도록 주의하고 조리가 완료된 음식은 실온에 보관하지 않아야 하며 특히 환기와 실내온도 관리에 유의하여야 한다. 앞으로 집단급식소의 조리실 설치 기준에 지하보다 지상의 환기 시스템이 잘 정비된 요건도 같이 마련되어야 하며, 해당 시설에서 환기 배관 시스템의 관리와 소독에 더 주의를 기울이도록 홍보할 필요가 있겠다.

**Acknowledgement**

심층 역학조사를 위해 현장에서 정보수집과 검체 채취를 해주신 제주도 제주시 제주보건소 송보나 주무관님과 제주시 위생관리과 김한솔 주무관님에게 감사를 표합니다.

**Conflict of interest**

The authors have no conflicts of interest to declare for this study.

**Funding**

None

**References**

1. Enosi Tuipulotu D, Mathur A, Ngo C, Man SM. *Bacillus cereus*: Epidemiology, virulence factors, and host-pathogen interactions. Trends Microbiol. 2021;29(5):458-71.
2. Dietrich R, Jessberger N, Ehling-Schulz M, Märtlbauer E, Granum PE. The food poisoning toxins of *Bacillus cereus*. Toxins (Basel). 2021;13(2):98.
3. Jovanovic J, Ornelis VFM, Madder A, Rajkovic A. *Bacillus cereus* food intoxication and toxicoinfection. Compr Rev Food Sci Food Saf. 2021 Jul;20(4):3719-3761.
4. Stenfors Arnesen LP, Fagerlund A, Granum PE. From soil to gut: *Bacillus cereus* and its food poisoning toxins. FEMS Microbiol Rev. 2008 Jul;32(4):579-606.
5. Ministry of Food and Drug Safety, Korea. Statistics for food poisoning.

[cited 2021 December 1]. Available from: https://www.foodsafetykorea.go.kr/portal/healthyfoodlife/foodPoisoningStat.do?menu_no=3724&menu_grp=MENU_NEW02 (Korean).

1. Kim JB, Jeong HR, Park YB, Kim JM, Oh DH. Food poisoning associated with emetic-type of *Bacillus cereus* in Korea. Foodborne Pathog Dis. 2010 May;7(5):555-63.
2. Korea Centers for Disease Control and Prevention. 2022 Guideline for controlling water- and food-borne infectious diseases. [cited 2022 May 16]. Available from: https://www.kdca.go.kr/board/board.es?mid=a20507020000&bid=0019 (Korean)
3. Glasset B, Herbin S, Guillier L, Cadel-Six S, Vignaud ML, Grout J, et al. *Bacillus cereus*-induced food-borne outbreaks in France, 2007 to 2014: epidemiology and genetic characterisation. Euro Surveill. 2016 Dec 1;21(48):30413.
4. Taylor JM, Sutherland AD, Aidoo KE, Logan NA. Heat-stable toxin production by strains of *Bacillus cereus*, *Bacillus firmus*, *Bacillus megaterium*, *Bacillus simplex* and *Bacillus licheniformis*. FEMS Microbiol Lett. 2005 Jan 15;242(2):313-7.
5. Agata N, Ohta M, Yokoyama K. Production of *Bacillus cereus* emetic toxin (cereulide) in various foods. Int J Food Microbiol. 2002 Feb 25;73(1):23-
